# The Relationship between Emotional Intelligence, Job Satisfaction, and Organizational Commitment among First-Line Nurse Managers in Qatar

**DOI:** 10.1155/2024/5114659

**Published:** 2024-07-25

**Authors:** Mutaz I. Othman, Anas Khalifeh, Islam Oweidat, Abdulqadir J. Nashwan

**Affiliations:** ^1^ Nursing Department Hamad Medical Corporation, Doha, Qatar; ^2^ Faculty of Nursing Zarqa University, Zarqa, Jordan

## Abstract

**Background:**

Emotional responses and the ability to regulate emotions among nurses, especially first-line nurse managers, can influence various workplace dynamics. However, there is a knowledge gap regarding emotional intelligence, job satisfaction, and organizational commitment among nurses in Qatar, particularly first-line nurse managers.

**Objectives:**

The primary aim of this study is to determine if there is a significant relationship between emotional intelligence, job satisfaction, and organizational commitment among first-line nurse managers (FLNMs) in Qatar.

**Design:**

This is a descriptive cross-sectional correlational study. *Settings*. The research took place at Hamad Medical Corporation in Qatar. *Participants*. A total of 203 first-line nurse managers participated in the study.

**Methods:**

Participants were recruited using a convenience sample method. Data were collected using the Genos Emotional Intelligence Inventory—Concise, the three-component model Employee Commitment Survey, and the short-form Minnesota Satisfaction Questionnaire (MSQ).

**Results:**

The findings showed that participants had average levels of emotional intelligence, job satisfaction, and organizational commitment. Notably, a significant, moderately positive relationship was observed between emotional intelligence and organizational commitment, as well as between job satisfaction and organizational commitment. A weak positive relationship was identified between emotional intelligence and job satisfaction. Differences in emotional intelligence were observed based on variables such as gender, age, and unit specialty. Organizational commitment varied based on the current position and hospital type, while job satisfaction differed based on the current position and education level.

**Conclusions:**

The findings suggest that enhancing the emotional intelligence of first-line nurse managers can potentially improve organizational commitment, job satisfaction, and, subsequently, healthcare outcomes. There is a need for further research to delve deeper into these factors and devise strategies aiming to boost the emotional intelligence and job satisfaction of first-line nurse managers. *Implications for Nursing Management*. The study provides empirical data from Qatar's healthcare system, shedding light on FLNMs' emotional intelligence, job satisfaction, and organizational commitment.

## 1. Introduction

Leadership is a major challenge for healthcare organizations and nurse managers in the 21st century, as dynamic and rapid organizational and environmental changes require the availability of effective managers at all organizational levels. Nurses are a crucial component of the healthcare system, and nurse managers play an important role in ensuring quality patient care [1].

First-line nurse managers (FLNMs) are registered nurses with a license who work in a managerial position in a healthcare organization, typically at the operational level. They are in the position of managing and supervising a team of nurses, along with ensuring the seamless operation of the nursing unit [2]. FLNMs play a key role in nurse success and patient outcomes [1]. Therefore, it is important for them to focus on improving retention rates and meeting performance expectations [3]. FLNMs' performance has a significant impact on job satisfaction and commitment [4]. Emotional intelligence (EI), on the other hand, has been shown to be an important factor in the workplace that affects job performance, job satisfaction, and organizational commitment [5]. The important role of EI in nursing comes from its impact on workplace harmony [6]. Also, EI involves controlling emotions, motivating others [7], fostering positive relationships, job satisfaction, and productivity as well [8, 9]. Healthcare managers along with FLNMs receive benefits from EI [9], FLNMs with EI influence quality of care, safety, staff nurse retention, and patient outcomes [10]. In contrast, a lack of EI in FLNMs leads to lower nurses', engagement, higher turnover, and lower staff nurse retention [11, 12]. As a result, EI fosters positive relationships and job satisfaction between nurses [5, 8].

Many FLNMs experience job-related stress due to insufficient knowledge and skills necessary for a challenging work setting [13]. Therefore, many FLNMs may not have been able to adapt to their position successfully.

FLNMs may lack knowledge and skills, causing stress and ineffective performance [13]. Incompetent FLNMs affects patient and staff nurses outcomes, so they required a competency-based and well-structured strategy for the development of FLNMs [2]. So, developing FLNMs with EI is crucial [2], but little study has been conducted on the EI of FLNMs [10]. EI can help retain and motivate FLNMs [14]. In general, it is believed that employees with more EI will perceive higher job satisfaction. This is due to the assumption that individuals with greater EI can develop strategies for dealing with potential stress-related consequences, while others with lower EI cannot. EI has been acknowledged as a crucial competency for healthcare professionals [4]. So, administrators and policymakers might use EI in healthcare as a strategy to develop organizational strategies for the retention and motivation of all FLNMs.

FLNMs play a vital role in nursing by bridging the gap between senior management and clinical nurses. The positive impact of enhancing EI and commitment among nurses and FLNMs leads to improved decision-making, care quality, and job satisfaction [15]. This will allow FLNMs to focus on building relationships and communicating well with nurses.

Job satisfaction, both intrinsic and extrinsic, refers to an employee's fulfillment with a particular job. The internal factors of an individual, such as personality and personal perspectives, as well as the exterior characteristics of the workplace, such as the work environment, can influence job satisfaction. Job characteristics can have an impact on an employee's wellbeing and affect job satisfaction and dissatisfaction [16]. Spector's definition of job satisfaction is one of the most common ones. It says job satisfaction depends on how a person feels about their job and all its parts [17].

An employee derives intrinsic satisfaction from the individual internal motivation when performing a certain task to achieve satisfaction, and as a result, satisfaction is derived from the staff member's active engagement in the assigned task [18–20]. On the other hand, extrinsic satisfaction derives from external elements such as money, promotion, and the anticipation of receiving rewards [21]. Theories on human motivation, such as Maslow's needs hierarchy theory, Herzberg's motivator-hygiene theory, the job characteristics model, and the dispositional approach, are associated with theories on job satisfaction. This is due to the close relationship between the two sets of concepts [21].

On the other hand, organizational commitment is defined as a person's connection with and involvement in his or her current organization. This includes an individual's acceptance of the organization's objectives and values, along with a strong wish to continue working for that organization [22]. Job satisfaction and commitment are distinct concepts. Such a being emotionally attached to the organization indicates a deep loyalty to the organization.

Organizational commitment indicates that an employee cares about the organization's goals and values, whereas job satisfaction focuses on the employee's job [23]. John Meyer and Natalie Allen developed the organizational commitment theory [24].

Commitment in the workplace can be categorized into the following three dimensions: affective, normative, and continuous [24]. Affective commitment involves an employee's emotional attachment and involvement in their job. Normative commitment is when an employee feels obligated to stay loyal to the organization, often due to perceived investments made by the company or a desire to support colleagues. Continuous commitment is driven by concerns about the costs and benefits associated with leaving the organization, such as loss of compensation or potential gains from staying in the job. Job satisfaction and commitment have significant effects on the performance of FLNMs [25].

This study set the stage for future studies on the relationship between EI, job satisfaction, and organizational commitment among FLNMs in Qatar and other countries, along with factors that may affect that relationship.

This study aimed to examine the relationship between EI, job satisfaction, and organizational commitment among FLNMs in Qatar. This study intends to assess the emotional intelligence, organizational commitment, and job satisfaction levels among first-line nurse managers in Qatar and, in addition, examine differences based on demographic and organizational characteristics, as well as the relationships among these variables.

## 2. Methods

### 2.1. Design of the Study and Data Collection

A descriptive cross-sectional design examined the relationship between EI, job satisfaction, and organizational commitment. While this design provides a clear snapshot at a specific time, it has limitations, including the inability to establish causality [26]. The study involved ethical approval, online questionnaire use, and recruitment via email from the nursing corporation office and research nursing department to all target sample emails attached with a cover letter and questionnaire link. The survey was accessible on various devices, and invitations were sent through the research department to ensure equal opportunity. The data collection procedure took a total of four weeks. It involved sending an email two weeks after the initial invitation and another reminder email three weeks later, and the survey took approximately 20 minutes to complete.

### 2.2. Participants and the Sampling Procedure

The target population for this study is FLNMs in Qatar. The accessible population was recruited from FLNMs among Hamad Medical Corporation (HMC) participants. HMC manages nine specialty hospitals and three community hospitals, and as well as being one of the largest employees in Qatar, HMC is the main healthcare provider in Qatar. Nonprobability convenience sampling was employed for those who met specific criteria, such as having managerial roles in the nurse and midwife career framework, holding a bachelor's degree, and working full time in selected hospitals for at least one year. The study used G*∗*Power 3.1.9.7 software to calculate the required sample size, resulting in a minimum of 138 using a correlation test (power = 0.95, alpha = 0.05, two-tailed, and effect size = 0.3).

### 2.3. Instruments

The first section covers sociodemographic and professional characteristics such as age, gender, job position, educational level, years of experience as a nurse, years of experience as a first-line nurse manager, and nursing specialty unit. Then, three instruments were used to collect data in this study, reflecting each main variable in the study: the EI Genos Emotional Intelligence Inventory-Concise (self-assessment). For the Genos EI Inventory, one must use a 5-point Likert scale with 31 items. A 5-point Likert scale contains 31 items: 1 = almost never, 2 = seldom, 3 = sometimes, 4 = usually, and 5 = almost always. The questionnaire is specific to a workplace setting and takes 5–7 minutes to complete. Genos' instrument was selected because it has sufficient face validity, is clear and understandable, and is accessible. The scoring involves summing up the scores from different scales: Emotional Self-Awareness, Emotional Expression, Emotional Awareness of Others, Emotional Reasoning, Emotional Self-Management, Emotional Management of Others, and Emotional Self-Control. Each scale includes specific items, and the total Emotional Intelligence score is calculated by adding up the scores from all these scales. Percentile scores are used for interpretation, representing the percentage of individuals scoring below a particular raw score. The Genos EI Inventory provides percentile ranges, categorizations, and interpretive guidelines for scoring and interpretation [27]. The Genos EI Inventory (Concise) Scale's alpha reliability coefficient has been determined by previous research to be 0.86, and its subscale reliability is good enough [28].

The job satisfaction score was calculated using the short-form Minnesota Satisfaction Questionnaire (MSQ), developed by Weiss, Dawis, England, and Lofquist in 1967. The questionnaire consists of 20 items rated on a 5-Likert scale from 1 (dissatisfied) to 5 (very satisfied). Two factors, intrinsic and extrinsic satisfaction, are derived from the factor analysis of the 20 items. The general satisfaction score can be calculated by combining these two elements. The intrinsic scale includes 12 items, while the extrinsic scale includes 6 items. Two items are part of the general satisfaction scale. Raw scores for each scale can be converted to percentile scores, with a score of 75 or higher indicating high satisfaction, a score of 25 or lower indicating low satisfaction, and a score between 26 and 74 indicating average satisfaction. The reliability coefficients for intrinsic satisfaction, extrinsic satisfaction, and general satisfaction are 0.86, 0.8, and 0.9, respectively [29].

The revised TCM Employee Commitment Survey by Meyer and Allen measures FLNM organizational commitment. It includes 18 items and six items measuring affective, continuance, and normative commitment [24]. The organizational commitment scores are calculated by computing averages for the Affective Commitment Scale (ACS), Normative Commitment Scale (NCS), and Continuance Commitment Scale (CCS) for each respondent. After reading each item, employees indicate the strength of their agreement by selecting a number from 1 (strongly disagree) to 7 (strongly agree). The implementation level of organizational commitment is determined by adding the range value to the level with the lowest mean score after obtaining the range value. Reports on reliability and validity show that this subscale has acceptable reliability ranging from 0.74 to 0.89 and construct validity via factor analysis confirming affective commitment as a distinct measure of organizational commitment [24]. This approach helps in effectively interpreting the data analysis and enhances the efficacy of data analysis and interpretation, which were computed using the subsequent formula [30]:

(1) $$\begin{array}{c} \text{Range Value}=\frac{\text{highest mean score}-\text{lowest mean score}}{\text{Number of level intended }}. \end{array}$$

A pilot study on 30 participants was conducted to assess survey clarity, filling time, analytic procedure, and participant response; the pilot subjects were not included in this study. Using Statistical Package for the Social Sciences (SPSS) Statistics version 26 software by IBM, the instrument's validity and reliability were evaluated using Cronbach's alpha coefficient, ensuring internal consistency and reliability. The instruments used for evaluation of Cronbach's alpha coefficient were the Genos Emotional Intelligence Inventory-Concise (self-assessment) (0.867), MSQ (0.939), and TCM Employee Commitment Survey (0.831). The study utilized English language instruments as the participants mainly spoke English.

### 2.4. Ethical Considerations

The study received ethical approval from Zarqa University and the Institutional Review Board (IRB) of the Medical Research Committee (MRC-01-23-058) at HMC. Email invitations were sent to participants with the assistance of the nursing corporate workforce. Participants were provided with an information sheet explaining the study, its risks and benefits, and their right to participate. Anonymity and privacy were maintained, and completed surveys were secured. Permission was obtained to use the research instruments.

### 2.5. Statistical Analysis

Statistical Package for the Social Sciences (SPSS) Statistics version 26 software by IBM was used for descriptive and inferential statistics. The data were coded, cleaned, and checked for normality assumptions. Data cleaning was performed to ensure the accuracy and reliability of the data. Frequencies and percentages describe sample characteristics. Independent *t*-tests and ANOVA tests examined differences in mean scores. Pearson's correlation explored relationships between variables.

## 3. Results

Out of 250 first-line nurse managers, 203 responded (81.2% response rate). As shown in Table 1, the majority were female (54.7%) and aged 35–44 years (61.6%). Most had a bachelor's degree (75.9%), while 24.1% had a master's degree. The majority had more than 10 years of nursing experience (83.2%) and more than 5 years as FLNMs (93.6%). Various positions, hospitals, and medical specialties are represented in Table 1.

The participants showed an average level of EI (*M* = 49.87 and SD = 28.89), with 42.4% above average and 41.4% below average, as shown in Table 2. The highest domain was emotional management of others (*M* = 3.99 and SD = 0.62), while the lowest was emotional expression (*M* = 3.48 and SD = 0.54), see Tables S1–S6 in the Supplementary Material for more details. As a result, as shown in Table 3, organizational commitment level averaged 4.55 (SD ± 0.83); on the other hand, affective commitment scoring was the highest (*M* = 4.72 and SD ± 1.22) and continuance commitment was the lowest (*M* = 4.42 and SD ± 1.05), as shown in Table S2. The result represented in Table 4 shows that job satisfaction level had a mean score of 68.13 (SD ± 12.51), with extrinsic satisfaction (*M* = 3.90 and SD ± 0.50) higher than intrinsic satisfaction (*M* = 3.47 and SD ± 0.58), as shown in Table S3.

On the other hand, the differences with sociodemographic and professional characteristics show that gender has a significant difference with EI (*t* = 2.134, *p* < 0.05), and age groups differ significantly (*F*(3,199) = 3.25, *p* < 0.05). Nursing unit specialties showed significant differences in EI (*F*(12,190) = 2.58, *p* < 0.05) also. On the other hand, current position, educational level, years of experience, and hospital type did not have a significant difference with EI, as shown in Table S4. While gender did not have a significant difference with organizational commitment (*t* = 1.43 and *p*=0.15), the current position has a significant difference (*F*(3,199) = 2.930, *p* < 0.05), with head nurses reporting higher commitment. Hospital type influenced organizational commitment (*F*(14,188) = 3.67, *p* < 0.05), with primary hospitals having a significant difference in organizational commitment as shown in Table S5. Job satisfaction did not significantly differ based on gender (*t* = 0.20, *p*=0.84), but educational level affected it (*t* = 2.29, *p* < 0.05), with bachelor's degree holders reporting higher satisfaction. The current position also affected job satisfaction (*F*(3,196) = 3.70, *p* < 0.05), with head nurses reporting higher satisfaction. Age group, years of experience, hospital type, and unit specialty did not have a significant difference with job satisfaction, as shown in Table S6. EI and organizational commitment had a moderate positive correlation (*r* = 0.319, *p* < 0.01), as EI and job satisfaction resulted in a weak positive correlation (*r* = 0.262, *p* < 0.01). While organizational commitment and job satisfaction show a moderately positive correlation (*r* = 0.599, *p* < 0.01), as shown in Table 5.

## 4. Discussion

This study was conducted to examine the relationship between emotional intelligence, organizational commitment, and job satisfaction among FLNMs in Qatar. Due to the dynamic nature of the healthcare sector and encountering new challenges, FLNMs' role in ensuring high-quality patient care and efficient operations has become increasingly crucial. The study revealed that FLNMs' EI level was average, which is consistent with findings from previous studies [15, 31–33]. However, some studies reported below-average levels of EI among FLNMs [34–36]. That may be because cultural and educational differences, workplace culture, job stress, and the workplace environment can influence EI. On other hand, according to the findings of this study, FLNMs exhibit a moderate to high level of organizational commitment [24, 37–40]. This may be attributable to their position within the organization, as they are linked to higher-level managers and administration, so they influence decision-making and have been perceived for their value in the organization. In addition, this may be attributable to the fact that they have been effective in the organization.

FLNMs' job satisfaction level was shown to be moderate to high [41–44]. In response to the study result, the instrument used in the instruction asked the participant to respond based on “how I feel about my current job.” Since the level of job satisfaction corresponds with EI, EI is the ability to recognize, understand, and control one's own emotions as well as the emotions of others. In contrast, job satisfaction is a feeling of satisfaction or fulfillment that an individual gives to his or her job. It makes sense to think that people with higher EI are better able to control their feelings at work and, as a result, are more likely to be satisfied with their jobs. Since EI is closely related to how professionals exercise their autonomy and manage their emotions, it makes sense that their EI would influence how they perceive and respond to these aspects of their job.

The study found that older FLNMs and those working in specialized units had higher EI scores than their peers. Also, research suggests that female tend to exhibit a higher level of EI in comparison to male. These findings are consistent with prior studies [45, 46]. EI is a skill that can be developed and enhanced through training and experience [47]. As FLNMs gain experience, they may develop greater EI skills that allow them to better understand and manage their own and others' emotions. A study conducted in Jordan to examine the relationship between emotional intelligence and nurse-nurse collaboration revealed that participants' gender, age, and years of experience were not significant factors in EI. Conversely, there were significant differences in EI based on nurse unit specialties and educational level [48]. Nursing personnel must be both curative and compassionate, especially during critical periods and end-of-life care, in line with the profession's core principles. This aspect of the work reinforces the performance-enhancing effects of EI on nurses. Gradually, this dependence on emotional intelligence may increase their awareness of their own emotional intelligence-related behaviors [36], allowing them to actively contribute to the provision of high-quality interdisciplinary care. Specifically, effective interactions between nurses and patients during end-of-life care could consistently foster a healing environment in the hospital. That is why you will see a difference in EI level based on age and nursing unit specialty.

The study found no significant association between emotional intelligence and level of education, current position, years of nursing experience, years of FLNM experience, or hospital type. These results are also consistent with previous studies [49, 50].

According to the results of this study, there was no significant difference in job satisfaction based on gender, age, hospital type, years of experience in the nursing field, years of experience as FLNM, or specialty. However, there were significant differences in job satisfaction based on current positions and levels of education.

A study examined job satisfaction among nurses in Egypt found no significant gender-based differences. These align with the study's findings [51]. The research also showed a significant difference in job satisfaction based on the educational level, with nurses holding a bachelor's degree reporting higher levels of job satisfaction than those with a master's degree, continuing to support the result.

In addition, a cross-sectional study on the job satisfaction of nurses in Saudi Arabia discovered that there were no significant differences based on gender, age, education level, or years of experience [52]. In another study, the authors investigated the relationship between organizational commitment and the job satisfaction of nurses in a Dubai hospital. They presented the results of their study and demonstrated the significance of different demographic factors, including age, education, years of experience, position, and specialization, on organizational commitment and job satisfaction [53]. One more study found that those with postbasic nursing education reported significantly greater job satisfaction than those with only basic education (*p* < 0.05) [54].

A cross-sectional study conducted with a random sample of 5000 licensed nurses in the United States found that nurses' levels of job satisfaction did not vary by age, gender, educational level, or years of experience, except for hospital type, which had a significant difference in job satisfaction (*p*=0.02), which also aligns with the study findings [55, 56]. According to the results of this study, there were significant differences in organizational commitment based on current position and hospital type. These findings are also consistent with prior studies.

A study investigated organizational commitment among nurses at a major public hospital in Saudi Arabia. Researchers revealed that participant gender, educational level, and years of nursing experience were not significant predictors of overall organizational commitment. Only age was a significant predictor of organizational commitment [57].

This research shows that the relative participation of males and females is not independent of other factors, given the issues in modern life, the equal gender combination in the workplace and organizations, and the social and cultural obligation for both males and females to participate in vocational activities. Therefore, other factors rather than gender affect FLNMs' organizational commitment.

Another study found that older nurses had higher level organizational commitment than younger nurses, nurses with higher education levels had higher organizational commitment than those with lower education levels, and nurse managers with more work experience had higher organizational commitment than those with less work experience [58].

Regarding hospital type, a multisite survey study was conducted and found no statistically significant differences in education level, current position, specialty, or hospital type [39]. One more study revealed a positive and significant association between organizational commitment and years of experience in the current position but no significant difference depending on age, gender, unit specialty, or educational level [59].

EI correlated positively with organizational commitment and job satisfaction [40, 56, 60–62]. FLNMs showed a positive relationship between organizational commitment and job satisfaction [53, 58, 63]. But it is important to remember that correlation does not necessarily indicate causation. However, it is possible that additional factors, such as salary, workload, or degree of job autonomy, may also influence their total satisfaction with their jobs.

The research findings have demonstrated that FLNM with high levels of emotional intelligence demonstrate an increased willingness to have positive attitudes towards their job and organization, leading to increased organizational commitment and job satisfaction. Furthermore, emotional intelligence can enhance FLNMs' understanding and control of their emotions, therefore promoting higher levels of job satisfaction and organizational commitment.

## 5. Limitations

There are a few limitations to this research. The current study's cross-sectional design is susceptible to usual biases, such as lower generalizability, including the inability to establish causality [26]. Furthermore, the implementation of self-assessment questionnaires could have generated self-report biases, such as “inflation” and “manipulation” biases. Indeed, the use of reliable and validated psychometric scales helped mitigate some of the self-report biases associated with self-assessment questionnaires. By employing established and well-tested instruments, researchers can enhance the credibility and accuracy of the data collected in the study. As an additional limitation, there could be other variables not included in the study that may influence the observed relationships. For instance, work-related stress, leadership style, or organizational support could have an impact on both emotional intelligence and job satisfaction. Furthermore, this limits the use of advanced methods of statistical analysis, such as linear regression.

## 6. Implications on Nursing Management

The study shows EI has a positive influence on job satisfaction and organizational commitment for FLNMs. Human resources should invest in EI training for FLNMs and consider it a core competency during recruitment. Nursing programs should invest in EI training programs and incorporate them into nursing curriculum content to prepare future nurses, while continuing education is essential. In addition, it is important to consider and incorporate these factors into nursing strategies and the policy-making process. Future research should prioritize conducting experimental studies to explore the potential impact of EI on job satisfaction and organizational commitment. It is important to consider a wide range of variables in these studies, including leadership, occupational stress, role stress, innovative behavior, social relations, and technology trends in the workplace.

Researchers should investigate the impact of emotional intelligence on the future of work and first-line nurse managers' perceptions of digital innovation in the workplace. By implementing these strategies, healthcare organizations can optimize their management practices and enhance workforce performance. Another variable that has been largely overlooked is the relationship between emotional intelligence and technology trends in the workplace.

## 7. Conclusion

This study highlights the importance of improving the emotional intelligence, job satisfaction, and organizational commitment of FLNMs in Qatar. Strategies include education, leadership development, and work culture. Implementing flexible work hours, recognition programs, and talent development can create a positive work environment, fostering job satisfaction and organizational commitment. Further research is needed to understand this relationship in different healthcare settings.

## Figures and Tables

**Table 1:** Sociodemographic and professional characteristics (*n* = 203).

Variables	Frequency (n)	Percentage (%)
Gender		
Male	92	45.3
Female	111	54.7
Age		
≤34	42	20.7
35–44	125	61.6
≥45	36	17.7
Educational level		
Bachelor	154	75.9
Master's	49	24.1
Experience in the nursing field		
6–10 year	34	16.8
11–15 years	90	44.3
16–20 years	41	20.2
>21 years	38	18.7
Experience as first-line nurse manager		
1–5 year	13	6.4
6–10 years	75	36.9
11–15 years	54	26.6
>16 years	61	30.1
Current position		
Head nurse	37	18.2
Charge nurse	79	38.9
Acting head nurse	16	7.9
Acting charge nurse	71	35
Type of hospital		
Primary hospital	66	32.5
Secondary hospital	34	16.8
Tertiary hospital	103	50.7
Nursing specialty unit		
Cardiology	7	3.4
Critical care	39	19.2
Emergency	13	6.4
Geriatric care	17	8.4
Surgical	26	12.8
Peri-operative	7	3.4
Operation theatre	6	3.0

**Table 2:** Level of emotional intelligence (*n* = 203).

Categorization	Frequency (*n*)	Percentage (%)
Very high	43	21.2
High	43	21.2
Average	33	16.2
Low	37	18.2
Very low	47	23.2

**Table 3:** Level of organizational commitment (*n* = 203).

Categorization	Frequency (*n*)	Percentage (%)
Very high	10	4.9
High	85	41.9
Moderate	93	45.8
Low	14	6.9
Very low	1	0.5

**Table 4:** Level of job satisfaction (*n* = 203).

Categorization	Frequency (*n*)	Percentage (%)
High job satisfaction	68	33.5
Middle job satisfaction	77	37.9
Low job satisfaction	58	28.6

**Table 5:** Relationship between emotional intelligence, organizational commitment, and job satisfaction.

Variables		Organizational commitment	Job satisfaction
EI	*r*	*0.319 * ^*∗*^	*0.262 * ^*∗*^
*p* value		*p* ≤ 0.001	*p* ≤ 0.001
OC	*r*		*0.599 * ^*∗*^
*p* value			*p* ≤ 0.001

*Note.* EI = emotional intelligence; OC = organizational commitment, ^*∗*^significant at the 0.01 level.

## Data Availability

The data that support the findings of this study are available from the corresponding author upon reasonable request.

## Supplementary Materials

Supplementary Materials See Tables S1–S6 in the Supplementary Material for comprehensive data analysis.
