# Supplementary material for: The Relationship between Emotional Intelligence, Job Satisfaction, and Organizational Commitment among First-Line Nurse Managers in Qatar
Source: J Nurs Manag. 2024 Jul 25;2024:5114659. doi: 10.1155/2024/5114659 (PMC11918854; doi:10.1155/2024/5114659)
Supplement: Supplementary Materials — See Tables S1–S6 in the Supplementary Material for comprehensive data analysis. [file 5114659.f1.docx]

| **Table S1.** *Mean and standard deviation Genos EI Subscales*   \| **Subscale** \| ***Mean*** \| ***SD*** \| \| --- \| --- \| --- \| \| **ESA** \| 3.85 \| ±0.63 \| \| **EE** \| 3.48 \| ±0.54 \| \| **EAO** \| 3.86 \| ±0.61 \| \| **ER** \| 3.75 \| ±0.62 \| \| **ESM** \| 3.76 \| ±0.54 \| \| **EMO** \| 3.99 \| ±0.62 \| \| **ESC** \| 3.88 \| ±0.62 \|   ESA=Emotional Self-Awareness, EE =Emotional Expression, EAO= Emotional Awareness of Others, ER=Emotional Reasoning, ESM=Emotional Self-Management, EMO=Emotional Management of Others, ESC=Emotional Self-Control. |
| --- | --- | --- | --- | --- | --- | --- | --- | --- | --- | --- | --- | --- | --- | --- | --- | --- | --- | --- | --- | --- | --- | --- | --- | --- |

| **Table S2.** *Mean and standard deviation Organizational Commitment Subscales*   \| **subscale** \| ***Mean*** \| ***SD*** \| \| --- \| --- \| --- \| \| **Affective** \| 4.72 \| ±1.22 \| \| **Continuance** \| 4.42 \| ±1.05 \| \| **Normative** \| 4.50 \| ±1.06 \| \| **Organizational Commitment** \| 4.55 \| ±0.83 \| |
| --- | --- | --- | --- | --- | --- | --- | --- | --- | --- | --- | --- | --- | --- | --- | --- |

| **Table S3.** *Mean and Standard Deviation of Short Form MSQ Subscales*   \| **subscale** \| ***Mean*** \| ***SD*** \| \| --- \| --- \| --- \| \| **Intrinsic satisfaction** \| 3.47 \| ± 0.58 \| \| **Extrinsic satisfaction** \| 3.90 \| ± 0.50 \| |
| --- | --- | --- | --- | --- | --- | --- | --- | --- | --- |

| **Table S4.** *The differences in emotional intelligence are based on sociodemographic and professional characteristics.*   \| **Variable** \| **Emotional Intelligence** \| \| \| \| --- \| --- \| --- \| --- \| \|  \| **Mean (SD)** \| **t or F** \| ***P*** \| \| Gender ^a^ \|  \| 2.134 \| 0.034* \| \| Male \| 45.13 (±29.61) \|  \|  \| \| Female \| 53.80 (±27.81) \|  \|  \| \| Age ^b^ \|  \| 4.88 \| 0.008* \| \| ≤ 34 \| 42.84 (±29.50) \|  \|  \| \| 35-44 \| 48.64 (±28.98) \|  \|  \| \| 45≥ \| 62.33(±24.4922) \|  \|  \| \| Educational Level ^a^ \|  \| 0.31 \| 0.57 \| \| Bachelor \| 49.22 (±29.16) \|  \|  \| \| Master’s \| 51.90 (±28.24) \|  \|  \| \| Experience in the nursing field Range (Years) ^b^ \|  \| 0.70 \| 0.58 \| \| 11-15 years \| 43.39 (±31.06) \|  \|  \| \| 16-20 years \| 50.81 (±30.59) \|  \|  \| \| 21-25 years \| 50.13 (±25.62) \|  \|  \| \| 26-30 years \| 52.72 (±27.76) \|  \|  \| \| >30 years \| 56.79 (±19.07) \|  \|  \| \| Experience as first-line nurse manager range(years) ^b^ \|  \| 1.07 \| 0.377 \| \| 1-5 year \| 39.54 (±33.37) \|  \|  \| \| 6-10 years \| 50.05 (±28.47) \|  \|  \| \| 11-15 years \| 48.80 (±26.73) \|  \|  \| \| 16-20 years \| 44.26 (±32.33) \|  \|  \| \| 21-25 years \| 57.87 (±30.63) \|  \|  \| \| 26-30 years \| 53.96 (±25.46) \|  \|  \| \| Current position ^b^ \|  \| 2.37 \| 0.07 \| \| Head Nurse \| 60.91(±30.68) \|  \|  \| \| Charge Nurse \| 46.95 (±28.06) \|  \|  \| \| Acting Head Nurse \| 51.51 (±24.89) \|  \|  \| \| Acting Charge Nurse \| 46.99 (±28.85) \|  \|  \| \| Type of hospital ^b^ \|  \| 1.44 \| 0.23 \| \| Primary Hospital \| 47.11 (±29.59) \|  \|  \| \| Secondary Hospital \| 57.26 (±27.94) \|  \|  \| \| Tertiary Hospital \| 49.20 (±28.62) \|  \|  \| \| Nursing Specialty Unit ^b^ \|  \| 2.24 \| 0.008* \| \| Cardiology \| 43.19(±33.59) \|  \|  \| \| Critical Care \| 43.74 (±28.81) \|  \|  \| \| Emergency \| 54.99 (±25.92) \|  \|  \| \| Geriatric Care \| 56.30 (±20.69) \|  \|  \| \| Surgical \| 60.32 (±22.81) \|  \|  \| \| Peri-Operative \| 57.65 (±23.14) \|  \|  \| \| Operation Theatre \| 46.96 (±23.84) \|  \|  \| \| OPD \| 35.32 (±36.37) \|  \|  \| \| Oncology \| 44.31(±35.68) \|  \|  \| \| Obstetrics And Gynecology \| 64.57 (±30.37) \|  \|  \| \| Medical \| 44.39 (±28.54) \|  \|  \| \| Mental health \| 89.97 (±7.25) \|  \|  \| \| Pediatrics \| 41.55 (±24.37) \|  \|  \| |
| --- | --- | --- | --- | --- | --- | --- | --- | --- | --- | --- | --- | --- | --- | --- | --- | --- | --- | --- | --- | --- | --- | --- | --- | --- | --- | --- | --- | --- | --- | --- | --- | --- | --- | --- | --- | --- | --- | --- | --- | --- | --- | --- | --- | --- | --- | --- | --- | --- | --- | --- | --- | --- | --- | --- | --- | --- | --- | --- | --- | --- | --- | --- | --- | --- | --- | --- | --- | --- | --- | --- | --- | --- | --- | --- | --- | --- | --- | --- | --- | --- | --- | --- | --- | --- | --- | --- | --- | --- | --- | --- | --- | --- | --- | --- | --- | --- | --- | --- | --- | --- | --- | --- | --- | --- | --- | --- | --- | --- | --- | --- | --- | --- | --- | --- | --- | --- | --- | --- | --- | --- | --- | --- | --- | --- | --- | --- | --- | --- | --- | --- | --- | --- | --- | --- | --- | --- | --- | --- | --- | --- | --- | --- | --- | --- | --- | --- | --- | --- | --- | --- | --- | --- | --- | --- | --- | --- | --- | --- | --- | --- | --- | --- | --- | --- | --- | --- | --- | --- | --- | --- | --- | --- | --- | --- | --- | --- | --- | --- | --- | --- | --- | --- | --- | --- | --- | --- | --- | --- | --- | --- | --- | --- |

| **Table S5.** *The differences in organizational commitment are based on sociodemographic and professional characteristics.* | | | |
| --- | --- | --- | --- |
| **Variable** | **Organizational Commitment** | | |
|  | **Mean (SD)** | **t or F** | ***P*** |
| Gender ^a^ |  | 1.43 | 0.15 |
| Male | 4.46(±0.82) |  |  |
| Female | 4.62 (±0.83) |  |  |
| Age ^b^ |  | 0.19 | 0.82 |
| ≤ 34 | 4.59 (±0.89) |  |  |
| 35-44 | 4.52(±0.77) |  |  |
| ≥45 | 4.59 (±0.95) |  |  |
| Educational Level ^a^ |  | 0.32 | 0.57 |
| Bachelor | 4.597 (±0.79) |  |  |
| Master’s | 4.39 (±0.92) |  |  |
| Experience in the nursing field Range (Years) ^b^ |  | 0.722 | 0.57 |
| 11-15 years | 4.54 (±0.77) |  |  |
| 16-20 years | 4.46 (±0.91) |  |  |
| 21-25 years | 4.59 (±0.75) |  |  |
| 26-30 years | 0.77(±0.17) |  |  |
| >30 years | 0.66 (±0.19) |  |  |
| Experience as first-line nurse manager age (years) ^b^ |  | 0.428 | 0.83 |
| 1-5 year | 4.55(±0.77) |  |  |
| 6-10 years | 4.47 (±0.82) |  |  |
| 11-15 years | 4.578 (±0.93) |  |  |
| 16-20 years | 4.75 (±0.73) |  |  |
| 21-25 years | 4.65 (±0.74) |  |  |
| 26-30 years | 4.45 (±0.74) |  |  |
| Current position ^b^ |  | 2.930 | 0.03* |
| Head Nurse | 4.84(±0.67) |  |  |
| Charge Nurse | 4.39 (±0.81) |  |  |
| Acting Head Nurse | 4.77 (±1.021) |  |  |
| Acting Charge Nurse | 4.52 (±0.86) |  |  |
| Type of hospital ^b^ |  | 3.67 | 0.03* |
| Primary Hospital | 4.74 (±0.76) |  |  |
| Secondary Hospital | 4.61 (±0.85) |  |  |
| Tertiary Hospital | 4.40 (±0.85) |  |  |
| Nursing Specialty Unit ^b^ |  | 1.30 | 0.21 |
| Cardiology | 4.84 (±0.81) |  |  |
| Critical Care | 4.46(±0.87) |  |  |
| Emergency | 4.53(±0.97) |  |  |
| Geriatric Care | 4.36(±0.98) |  |  |
| Surgical | 4.53(±0.84) |  |  |
| Peri-Operative | 5.00(±0.65) |  |  |
| Operation Theatre | 4.19(±0.40) |  |  |
| OPD | 4.57(±0.69) |  |  |
| Oncology | 4.27 (±0.78) |  |  |
| Obstetrics And Gynaecology | 4.61(±0.88) |  |  |
| Medical | 5.56(±0.61) |  |  |
| Mental health | 4.46 (±0.56) |  |  |
| Paediatrics | 4.54(±1.08) |  |  |
| Note. ^a^= Independent *t*-test; ^b^=ANOVA test; SD=Standard deviation; * Significant at the 0.05 level; ** Significant at the 0.01 level | | | |

| **Table S6.** *The differences in Job satisfaction based on Sociodemographic and Professional characteristics.* | | | |
| --- | --- | --- | --- |
| **Variable** | **Job Satisfaction** | | |
|  | **Mean (SD)** | **t or F** | **P** |
| Gender ^a^ |  | 0.20 | 0.84 |
| Male | 51.98(±3 30.3) |  |  |
| Female | 51.134(±29.62) |  |  |
| Age Range (years) ^b^ |  | 1.20 | 0.30 |
| ≤ 34 | 51.75 (±31.19) |  |  |
| 35-44 | 49.50 (±29.65) |  |  |
| ≥45 | 58.26 (±28.84) |  |  |
| Educational Level a |  | 2.29 | 0.02* |
| Bachelor | 54.21 (±29.14) |  |  |
| Master’s | 43.08(±30.84) |  |  |
| Experience in the nursing field Range (Years) ^b^ |  | 1.33 | 0.26 |
| 11-15 years | 53.22 (±25.57) |  |  |
| 16-20 years | 55.40 (±30.36) |  |  |
| 21-25 years | 49.78 (±30.09) |  |  |
| 26-30 years | 47.87 (±30.68) |  |  |
| >30 years | 44.14(±31.41) |  |  |
| Experience as first-line nurse manager range(years) ^b^ |  | 0.55 | 0.73 |
| 1-5 year | 53.04 (±23.39) |  |  |
| 6-10 years | 55.09 (±30.61) |  |  |
| 11-15 years | 51.43 (±28.83) |  |  |
| 16-20 years | 46.31 (±28.37) |  |  |
| 21-25 years | 45.44 (±32.22) |  |  |
| 26-30 years | 44.08 (±30.79) |  |  |
| Current position ^b^ |  | 3.70 | 0.01* |
| Head Nurse | 63.90 (±27.23) |  |  |
| Charge Nurse | 45.31 (±31.09) |  |  |
| Acting Head Nurse | 58.27 (±29.44) |  |  |
| Acting Charge Nurse | 50.46(±28.16) |  |  |
| Type of hospital ^b^ |  | 2.72 | 0.07 |
| Primary Hospital | 54.57 (±28.41) |  |  |
| Secondary Hospital | 59.29 (±31.93) |  |  |
| Tertiary Hospital | 47.00 (±29.58) |  |  |
| Nursing Specialty Unit ^b^ |  | 1.11 | 0.34 |
| Cardiology | 48.93 (±29.05) |  |  |
| Critical Care | 48.10 (±29.42) |  |  |
| Emergency | 49.89 (±36.50) |  |  |
| Geriatric Care | 54.41 (±32.87) |  |  |
| Surgical | 50.65 (±29.04) |  |  |
| Peri-Operative | 78.65 (±13.21) |  |  |
| Operation Theatre | 38.40 (±32.17) |  |  |
| OPD | 54.69 (±27.39) |  |  |
| Oncology | 34.56 (±28.98) |  |  |
| Obstetrics And Gynecology | 43.82 (±34.043) |  |  |
| Medical | 56.90 (±27.34) |  |  |
| Mental health | 62.41(±30.43) |  |  |
| Pediatrics | 47.29 (±31.75) |  |  |
| Note. a= Independent t-test; b=ANOVA test; SD=Standard deviation; * Significant at the 0.05 level; ** Significant at the 0.01 level. | | | |
